# Hybrid PET/MRI Imaging of 18F‐Fluorodeoxyglucose and 18‐kDa Translocator Protein for Presurgical Localization in Refractory Epilepsy

**DOI:** 10.1111/cns.70251

**Published:** 2025-02-06

**Authors:** Siqi Zhang, Jie Hu, Zhigang Qi, Chenyang Yao, Bixiao Cui, Jingjuan Wang, Zhenming Wang, Jie Lu

**Affiliations:** ^1^ Department of Radiology and Nuclear Medicine Xuanwu Hospital Capital Medical University Beijing China; ^2^ Department of Radiation Oncology, Xuanwu Hospital Capital Medical University Beijing China

**Keywords:** fluorodeoxyglucose, PET/MRI, refractory epilepsy, translocator protein 18 kDa

## Abstract

**Purpose:**

Surgery remains the only curative option for a third of refractory epilepsy patients, though success depends on precise localization of the epileptogenic zone (EZ). This study aims to assess the clinical value of hybrid ^18^F‐FDG and ^18^F‐DPA‐714 PET/MRI for accurate localization and precise boundary delineation.

**Methods:**

The refractory epilepsy patients who underwent surgery at Xuanwu Hospital from November 2022 to November 2023 were retrospectively recruited. Preoperative simultaneous ^18^F‐FDG and ^18^F‐DPA‐714 PET/MRI imaging were analyzed using the asymmetry index (AI) and a 4‐point visual score, with the surgical site and pathological findings serving as the gold standard.

**Results:**

A total of 43 patients (mean age: 26.30 ± 8.37 years, male: 28) were included in this study. Lesion localization accuracy within the EZ was 76.7% for ^18^F‐FDG PET/MRI, 69.8% for ^18^F‐DPA‐714 PET/MRI, and 60.5% for conventional MRI (*p* = 0.26). In 26 MRI‐positive cases, conventional MRI accurately localized all lesions within the EZ, with three cases showing negative findings on ^18^F‐FDG images and six on ^18^F‐DPA‐714. Among 17 MRI‐negative patients, thirteen demonstrated positive results on hybrid PET/MRI. Additionally, ^18^F‐DPA‐714 PET/MRI proved more effective in delineating lesion boundaries. Compared to ^18^F‐FDG, the AI score was significantly lower (0.25 ± 0.18 vs. 0.46 ± 0.19, *p* < 0.001), while the visual score was higher (4.00 ± 2.00 vs. 3.00 ± 0.00, *p* = 0.01).

**Conclusion:**

^18^F‐DPA‐714 PET/MRI can effectively complement conventional MRI in the preoperative assessment of refractory epilepsy, with localization accuracy on par with ^18^F‐FDG and enhanced capability in delineating lesion boundaries.

## Graphical abstract

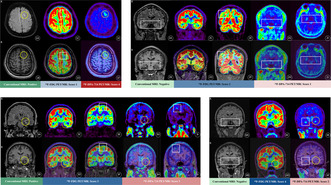

## 1 Introduction

Epilepsy, a prevalent chronic neurological disorder affecting approximately 65 million people globally [1], is the third leading contributor to neurological disease burden [2, 3]. Despite advances in antiseizure medications, about 30% of patients continue to experience seizures, termed refractory epilepsy, for which surgery is still the only curative option [4]. Accurate localization of the epileptogenic zone (EZ) is crucial for surgical success yet remains a significant challenge [5, 6].

Noninvasive localization of the EZ is fundamental for managing refractory epilepsy. While electroencephalogram (EEG) records electrophysiological activity, seizures' unpredictable timing and duration lead to variability and misdiagnosis [7]. MRI can identify lesions causing epilepsy, but despite advancements, it fails to provide adequate localization in nearly 30% of patients [8]. ^18^F‐FDG PET improves the detection of epileptogenic focus, especially in MRI‐negative patients, by highlighting hypometabolic regions [9, 10]. Nevertheless, these hypometabolic zones often extend beyond the true EZ, involving distant brain areas and thus compromising specificity [11, 12].

The novel PET radiotracer ^18^F‐DPA‐714, targeting the translocator protein 18 kDa (TSPO) on activated glial cells and astrocytes [13], has emerged as a promising biomarker for neuroinflammation [14] in identifying the EZ [15]. Evidence from preclinical research and limited human imaging studies [12, 16, 17, 18] indicates focal increases in TSPO PET within epileptogenic substrates. Still, due to small sample sizes, uncertainties persist regarding its specificity, detection capacity in extratemporal lobe epilepsy, and potential advantages over ^18^F‐FDG PET [19].

Positron emission tomography/magnetic resonance imaging (PET/MRI) enables the simultaneous acquisition of functional and structural data in a single scan, enhancing patient comfort and minimizing motion artifacts [20]. The current study aimed to compare (a) the accuracy of EZ detection and (b) the precision of border delineation between conventional MRI, ^18^F‐FDG PET, and ^18^F‐DPA‐714 PET/MRI.

## 2 Materials and Methods

### 2.1 Participants

We identified patients by reviewing the dual‐tracer simultaneous PET/MRI examination datasets from the past 2 years (2022.11–2024.11). Each patient provided informed consent for this retrospective observational study, which the Xuanwu Hospital Ethics Board approved (National clinical trial number: NCT06092125). Clinical factors, including age, sex, age at seizure onset, epilepsy duration, age at surgery, whether stereoelectroencephalography (SEEG) was implanted, surgical location, and pathological results, were reviewed.

Inclusion criteria were as follows: (1) Clinically diagnosed as refractory epilepsy, with comprehensive baseline clinical data and electrophysiological information; (2) The location of the EZ was confirmed through resection surgery outcomes; (3) Complete structural MRI, ^18^F‐FDG, and ^18^F‐DPA‐714 PET/MRI imaging were available. Patients were excluded for (1) Secondary epilepsy caused by autoimmune encephalitis or other etiologies, (2) diffuse structural abnormalities on MRI appearance, and (3) unsatisfactory imaging quality (e.g., severe image artifacts due to head movement).

### 2.2 PET/MRI Acquisition

PET/MRI exams were performed on an integrated simultaneous PET/MRI scanner (Signa GE Healthcare or uPMR790 United Imaging). The blood glucose level was < 150 mg/dL at the time of FDG injection. Video surveillance was employed to monitor the subjects to ensure that ^18^F‐FDG was administered neither during an ictal nor a postictal state. Following the injection of 3.7 MBq/kg of FDG, the subjects rested for 40 min in a quiet room to facilitate tissue uptake. For the ^18^F‐DPA‐714 tracer, 0.1–0.2 mCi/kg (1 mCi = 37 × 10^6^ Bq) was injected intravenously, followed by a 60‐min dynamic acquisition. The two PET/MRI acquisitions were performed for each patient within 2 weeks. All hybrid PET/MRI data were acquired following the previously standardized procedure [21, 22].

### 2.3 Imaging Analysis

#### 2.3.1 Overall Process

The conventional MRI and PET/MRI images were analyzed by two experienced radiologists certified in nuclear medicine and radiology. In case of discrepancies between their assessments, a chief imaging physician reviewed the results. Initially, the radiologists were blinded to the seizure symptoms, EEG findings, and surgical outcomes. Their task was to identify abnormalities for determining the predefined EZ and calculate the asymmetry index (AI). Subsequently, they evaluated the accuracy of the predefined EZ using surgical results as the gold standard, assigning a 4‐point visual score and categorizing patients into two subgroups: positive (3, 4) and negative (1, 2).

#### 2.3.2 Routine MRI Visual Assessment

Diagnostic criteria include MRI structural or signal abnormalities such as thickening or thinning of the local cerebral cortex, indistinct gray matter boundaries, abnormal cortical or subcortical signals, lobe atrophy or widening of sulci, and reduced hippocampal or temporal lobe volume.

#### 2.3.3 PET/MRI Asymmetry Index

The AI was used to evaluate the suspected epileptic zone in PET/MRI image analysis. In ^18^F‐FDG, anomalies were marked by areas with decreased tracer uptake, while in ^18^F‐DPA‐714, they were identified by areas with increased tracer uptake. The AIs for ^18^F‐FDG and ^18^F‐DPA‐714 were calculated from standardized uptake values using the formula: [200% × (ipsilateral − contralateral)/(ipsilateral + contralateral)] [11]. A lesion was considered questionable if the AI was greater than 10%.

#### 2.3.4 PET/MRI Visual Scores

Lesion boundaries were evaluated by observers using a 4‐point visual score based on a modified Paldino et al. [23, 24] method. The scoring criteria for EZ detection were as follows: Score 4 indicated clear detection of both EZ laterality and borders; Score 3 indicated clear laterality but slightly obscure borders; Score 2 indicated possible laterality with unclear borders; and Score 1 indicated poor detection of both laterality and borders. To facilitate statistical analysis, the patients were further divided into two subgroups: those with scores of 3–4 were classified as positive, and those with scores of 1–2 were classified as negative.

### 2.4 Statistical Analysis

Data were analyzed using SPSS software (IBM SPSS Statistics, Version 20.0) and GraphPad Prism software (Version 8.0.2). The normality of continuous parameter distributions was assessed using the Kolmogorov–Smirnov test. Normally distributed data were described as mean (standard deviation), while non‐normally distributed data were expressed as median (interquartile range). Categorical variables were expressed as proportions. A two‐sample *t*‐test or independent‐sample Mann–Whitney *U* test was used to compare continuous variables. The chi‐square test, Fisher's exact test, or the Wilcoxon rank‐sum test was used for categorical variables. A *p*‐value of < 0.05 was considered to be statistically significant.

## 3 Results

### 3.1 Study Population

According to the study criteria, the cohort comprised 43 patients (28 males and 15 females), with a mean age of 26.30 ± 8.37 years at the time of surgery. The mean disease duration was 13.31 ± 9.50 years. SEEG was implanted in 25 (58%) patients. All patients underwent lesion resection surgery, with Table 1 summarizing the distribution of EZs and pathologies. The EZs identified through surgery were confined to a single cerebral lobe, with 26 cases originating in the left hemisphere and 17 in the right. Statistical analysis indicated lesion localization: five patients in the frontal lobe, one in the parietal lobe, 23 in the temporal lobe, and four in the occipital lobe.

**TABLE 1:** Distribution of seizure origins based on surgical location and associated pathological findings.

Location	Subjects	Left	Right
Frontal lobe	5	4 [2 FCD, 2 NL]	1 [1 FCD]
Parietal lobe	1	1 [1 NL]	0
Temporal lobe	33	19 [4 FCD, 3 HS, 7 DP, 5 NL]	14 [3 FCD, 3 HS, 3 DP, 4 NL, 1 GG]
Occipital lobe	4	2 [1 FCD, 1 PA]	2 [1 FCD, 1 NL]

Abbreviations: DP, dual pathology (focal cortical dysplasia and hippocampus sclerosis); FCD, focal cortical dysplasia; GG, ganglioglioma; HS, hippocampus sclerosis; NL, nonlesional; PA, pilocytic astrocytoma.

### 3.2 PET/MRI Improves Visual Assessment

**FIGURE 1:**
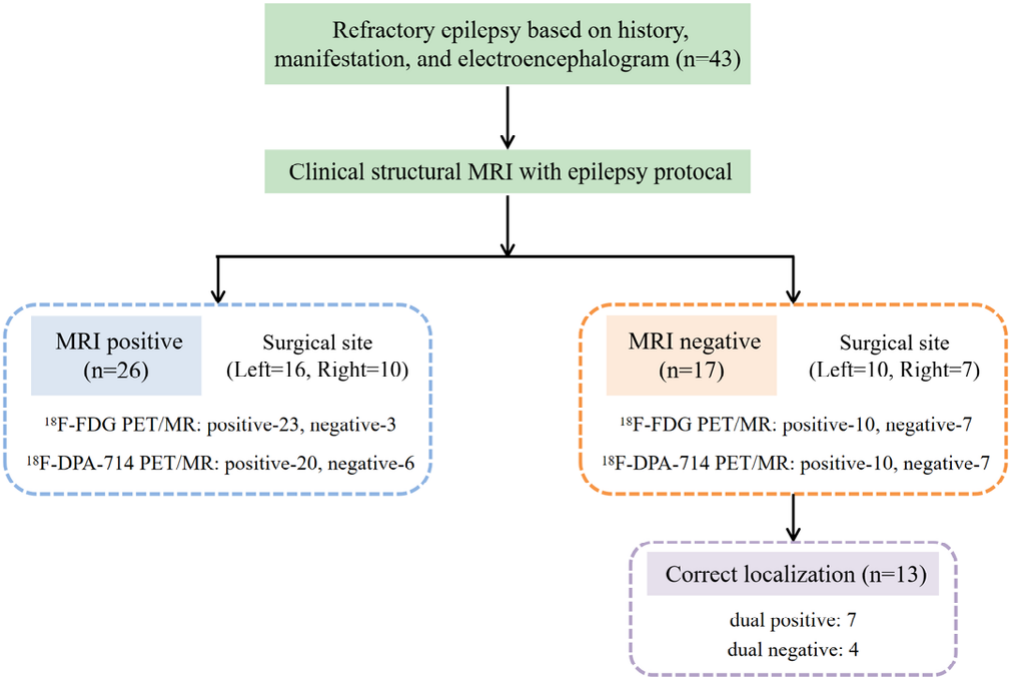
The flowchart of radiological assessment. Correct localization: MRI‐negative patients with positive findings on hybrid PET/MRI, and results consistent with the surgical resection localization.

Of the 43 patients, 17 (39.5%) had no or only subtle visually identifiable lesions, which classified them as MRI‐negative. PET/MRI imaging outperformed structural MRI in anomaly detection, with sensitivity rates of 76.7% for 18F‐FDG and 69.8% for ^18^F‐DPA‐714, compared to 60.5% for MRI (*p* = 0.26). In the 26 MRI‐positive cases, structural MRI accurately localized all lesions within the EZ, as confirmed by the surgical gold standard. However, ^18^F‐FDG PET/MRI failed to localize or lateralize lesions in 3 cases, while ^18^F‐DPA‐714 PET/MRI showed similar limitations in 6 cases. Notably, among the 17 MRI‐negative patients, 13 showed positive findings on hybrid PET/MRI, with ^18^F‐FDG and ^18^F‐DPA‐714 PET/MRI each accurately localizing lesions in 10 cases (58.8%). Further details are shown in Figure 1.

### 3.3 ^18^F‐DPA‐714 PET/MRI Enhances Border Delineation

**FIGURE 2:**
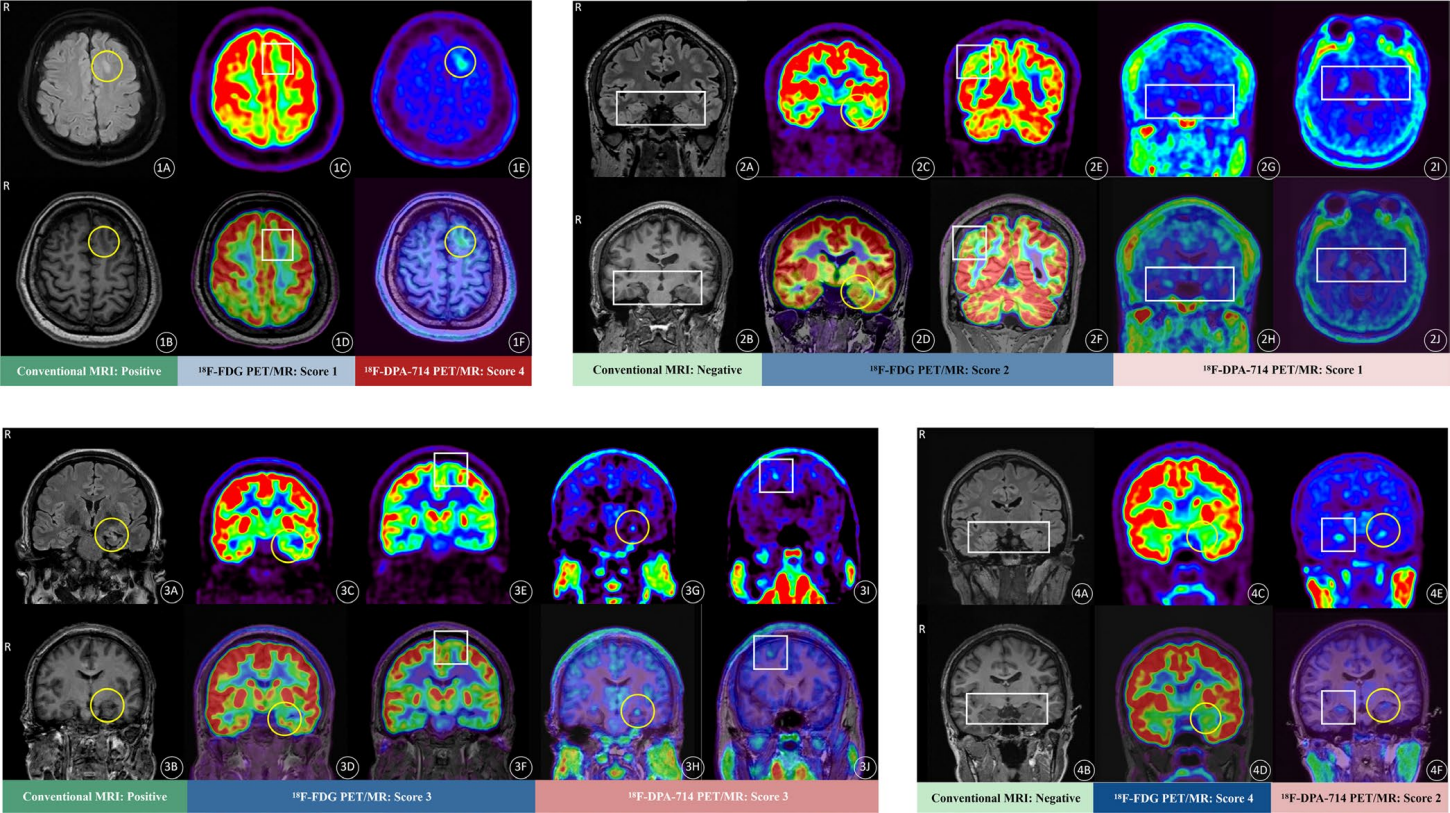
The 4‐point visual score evaluation of ^18^F‐FDG and ^18^F‐DPA‐714 PET/MRI. A 35‐year‐old female with a 29‐year history of epilepsy (Fig ①A–①F). Conventional MRI revealed localized cortical thickening and increased FLAIR signal in the left frontal lobe (yellow circle, Fig ①A, ①B), categorized as MRI positive. ^18^F‐FDG PET/MRI showed no significant metabolic reduction in the left frontal lobe (visual score 1, white rectangle, Fig ①C, ①D), indicating a negative result. ^18^F‐DPA‐714 PET/MRI demonstrated significantly increased TSPO uptake in the left frontal lobe, with an AI value of 0.52 and a visual score 4 (yellow circle, Fig ①E, ①F), categorized as positive. Postoperative histopathology confirmed left frontal lobe focal cortical dysplasia (FCD) IIb. A 22‐year‐old male with a 14‐year history of epilepsy (Fig ②A–②J). Conventional MRI showed no significant abnormalities (white rectangle, Fig ②A, ②B), categorized as MRI negative. ^18^F‐FDG PET/MRI revealed marked asymmetry in glucose metabolism reduction in the left temporal lobe (yellow circle, AI = 0.30, Fig ②C, ②D) and right parietal lobe (white rectangle, Fig ②E, ②F), with a visual score of 2 and categorized as negative. ^18^F‐DPA‐714 PET/MRI demonstrated increased uptake in both hippocampi without significant laterality (white rectangle, AI = 0.08, Fig ②G–②J), visual score 1, and categorized as negative. Postoperative histopathology confirmed FCD in the left temporal lobe and hippocampus. A 31‐year‐old male with a 4‐year history of epilepsy (Fig ③A–③J). Conventional MRI revealed atrophy and increased FLAIR signal in the left hippocampus (yellow circle, Fig ③A, ③B), categorized as MRI positive. ^18^F‐FDG PET/MRI showed marked asymmetry of glucose metabolism reduction within the left temporal lobe (yellow circle, AI = 0.50, Fig ③C, ③D) and frontal lobe (white rectangle, Fig ③E, ③F), with a visual score of 3 and categorized as positive. ^18^F‐DPA‐714 PET/MRI demonstrated marked asymmetry of increased TSPO uptake between the left temporal lobe (yellow circle, AI = 0.16, Fig ③G, ③H) and right frontal lobe (white rectangle, Fig ③I, ③J), with a visual score of 3 and categorized as positive. Postoperative histopathology confirmed dual pathology in the left temporal lobe: Hippocampal sclerosis combined with FCD. A 23‐year‐old female with a 9‐year history of epilepsy (Fig ④A–④F). Conventional MRI showed no significant abnormalities (white rectangle, Fig ④A, ④B), categorized as MRI negative. ^18^F‐FDG PET/MRI revealed marked asymmetry of glucose metabolism reduction within the left temporal lobe (yellow circle, AI = 0.24, Fig ④C, ④D), with a visual score 4 and categorized as positive. ^18^F‐DPA‐714 PET/MRI demonstrated increased TSPO uptake in both hippocampi, more pronounced on the right side (white rectangle for right, yellow circle for left, AI = 0.26, Fig ④E, ④F), visual score 2, and categorized as negative. Postoperative histopathology confirmed FCD in the left frontal lobe.

A lesion was considered questionable if the AI was greater than 10%, with 35 (81.4%) identified on ^18^F‐DPA‐714 PET/MRI and 42 (97.7%) on ^18^F‐FDG. The AI score for ^18^F‐DPA‐714 PET/MRI was lower than that for ^18^F‐FDG (0.25 ± 0.18 vs. 0.46 ± 0.19; *t* = 5.26, *p* < 0.001). Lesions with scores of 3–4 were classified as positive, and those with scores of 1–2 as negative, resulting in 30 (69.8%) positive cases on ^18^F‐DPA‐714 PET/MRI and 33 (76.7%) on ^18^F‐FDG. Furthermore, the visual score for ^18^F‐DPA‐714 PET/MRI was higher than that for ^18^F‐FDG (4.00 ± 2.00 vs. 3.00 ± 0.00; *z* = −2.47, *p* = 0.01). Individual records and representative examples of radiological assessments are presented in Table 2 and Figure 2.

**TABLE 2:** Comparison of the quantitative analysis of the 43 patients.

	^18^F‐FDG PET/MR (*n* = 43)	^18^F‐DPA‐714 PET/MR (*n* = 43)	*p* ^a^
Asymmetry index	0.46 ± 0.19	0.25 ± 0.18	< 0.001^b^
AI positive	42 (97.7)	35 (81.4)	0.03^b^
AI negative	1 (2.3)	8 (18.6)	
Visual scores			
4	9 (20.9)	27 (62.8)	0.01^b^
3	24 (55.8)	3 (7.0)	
2	4 (9.3)	10 (23.2)	
1	6 (14.0)	3 (7.0)	

*Note:* Data are presented as mean ± SD for normally distributed continuous variables and counts (percentages) for categorical variables.

^a^ A two‐sample *t*‐test was used for normally distributed continuous variables. The chi‐square test was used for categorical variables, Fisher's exact test for nonordinal categorical variables, and the Wilcoxon rank‐sum test for ordinal categorical variables.

^b^ Statistically significant, *p* < 0.05.

## 4 Discussion

In this original study, we compared the diagnostic performance of ^18^F‐FDG and ^18^F‐DPA‐714 PET/MRI in refractory epilepsy, with two main findings: (1) the diagnostic accuracy of ^18^F‐DPA‐714 PET/MRI was noninferior to that of ^18^F‐FDG for localizing seizure foci, and (2) ^18^F‐DPA‐714 PET/MRI provided stronger evidence for delineating EZ boundaries than ^18^F‐FDG. These findings confirm the clinical value of ^18^F‐DPA‐714 PET/MRI, particularly for MRI‐negative patients.

Both qualitative and quantitative visual analyses of ^18^F‐FDG and ^18^F‐DPA‐714 PET/MRI demonstrated robust sensitivity and accuracy. In a relatively large dataset (*n* = 43, compared to prior studies with *n* = 11–27) and with a considerable proportion of MRI‐negative cases (40% vs. 33%–65%) [12, 16, 17, 18, 25], we confirmed increased ^18^F‐DPA‐714 uptake in the EZ of patients with drug‐resistant epilepsy. For certain cases with pronounced structural abnormalities on conventional MRI but low side‐to‐side binding asymmetry indices, widespread inflammation in drug‐resistant epilepsy likely accounts for the results [16, 26]. This further highlights the importance of structural MRI in localization assessments while showcasing the advantages of integrated PET/MRI. Simultaneous multimodal data acquisition and analysis enrich the information available to clinicians and radiologists, offering a more comprehensive view.

The AI metric has been widely used in previous TSPO imaging studies [17, 18, 25], and we included it here due to its practicality and broad clinical application as a semi‐quantitative evaluation method. Nonetheless, in cases of bilateral anomalies without a clear side predominance, interpreting the AI as normal may be misleading, potentially leading to conclusions of either bilateral epileptogenicity or absent fixation in these regions. Voxel‐wise analysis, while beneficial for bypassing anatomical constraints, may be less effective due to its focus on individual rather than group‐based analysis in epilepsy, as each patient presents unique characteristics. Additionally, potential age and sex mismatches within the ^18^F‐DPA‐714 group may have affected the sensitivity of this method [12].

To address these challenges, we employed the 4‐score modified Paldino et al. approach [23], using surgical and pathological outcomes as the gold standard to further assess the precision of ^18^F‐FDG and ^18^F‐DPA‐714 PET/MRI in accurately delineating lesion boundaries. In visual scoring, ^18^F‐DPA‐714 PET/MRI demonstrated greater effectiveness than ^18^F‐FDG in detecting abnormal tracer uptake at lesion boundaries, with a score‐4 proportion of 63% versus 21%. The relatively well‐defined lesion area and higher specificity of ^18^F‐DPA‐714 uptake provide critical support for preoperative planning in clinical surgical resection. By providing a more precise delineation of the epileptogenic focus, this imaging modality could assist surgeons in more accurately targeting resection areas, potentially minimizing the risk of postoperative complications. Additionally, the enhanced precision could lead to improved postoperative outcomes, such as a higher likelihood of achieving seizure freedom, and contribute to a more personalized treatment approach for patients with refractory epilepsy. Nonetheless, this hypothesis required further validation in studies with larger sample sizes and prospective designs.

A limitation of our study was the absence of genotyping for the TSPO gene rs6971 polymorphism, which prevents differentiation between individuals with high, mixed, and low TSPO affinity. Given prior studies [27, 28] showing that most East Asians are high‐affinity binders (HABs) for TSPO, our findings may be more representative of this population. Moreover, nonspecific uptake regions, such as the pituitary and midbrain [18], were observed in all enrolled patients, suggesting that TSPO affinity in our cohort was relatively high, though individual variability in binding affinity was unexamined. Furthermore, due to the limited sample size, subgroup analyses based on epilepsy subtypes (e.g., temporal lobe epilepsy vs. extra‐temporal lobe epilepsy) or different pathological types of refractory epilepsy were not performed, as these may exhibit distinct TSPO uptake patterns. These aspects warrant further investigation in larger studies.

## 5 Conclusion

This study provides preliminary evidence that ^18^F‐DPA‐714 PET/MRI may offer significant supplementary value in the presurgical evaluation of drug‐resistant focal epilepsy. With greater specificity than ^18^F‐FDG, it could serve as a valuable tool for localizing the epileptogenic focus and refining lesion boundary delineation.

## Author Contributions

Conceptualization: S.Z.; Methodology: S.Z. and Z.Q.; Software: S.Z.; Validation: J.H. and C.Y.; Formal analysis: S.Z.; Investigation: J.H. and C.Y.; Resources: B.C. and Z.W.; Data curation: B.C. and Z.W.; Writing – original draft preparation: S.Z.; writing – review and editing: J.W. and J.L.; Visualization: S.Z.; Supervision: J.L.; Project administration: J.L.; Funding acquisition: J.L. All authors read and approved the final manuscript.

## Ethics Statement

The studies involving human participants were reviewed and approved by the Medical Ethics Committee of the Xuanwu Hospital (NO.2023044).

## Consent

Consent to participate: Informed consent was obtained from all individual participants included in the study.

Consent to publish: The authors have nothing to report.

## Conflicts of Interest

The authors declare no conflicts of interest.

## Data Availability

The datasets used and analyzed during the current study are available from the corresponding author upon reasonable request.
